# A Critique of Olfactory Objects

**DOI:** 10.3389/fpsyg.2019.01337

**Published:** 2019-06-12

**Authors:** Ann-Sophie Barwich

**Affiliations:** Cognitive Science Program, Indiana University, Bloomington, IN, United States

**Keywords:** olfaction, perceptual objects, sensory coding, odor receptors, chemoreception, stimulus representation, perceptual constancy, figure-ground segregation

## Abstract

Does the sense of smell involve the perception of odor objects? General discussion of perceptual objecthood centers on three criteria: stimulus representation, perceptual constancy, and figure-ground segregation. These criteria, derived from theories of vision, have been applied to olfaction in recent philosophical debates about psychology. An inherent problem with such framing of olfactory objecthood is that philosophers explicitly ignore the constitutive factors of the sensory systems that underpin the implementation of these criteria. The biological basis of odor coding is fundamentally different from the coding principles of the visual system. This article analyzes the three measures of perceptual objecthood against the biological background of the olfactory system. It contrasts the coding principles in olfaction with the visual system to show why these criteria of objecthood fail to be instantiated in odor perception. The argument demonstrates that olfaction affords perceptual categorization without the need to form odor objects.

## Theories of Perception ≠ Theories of Vision

Theories of perception suffer from one fundamental flaw: they are theories of vision. What gets routinely referred to as “the other senses” hardly played a role in general discussions about the nature of sensory perception, until recently. Systematic bias toward vision as the paradigm sense continues to shape both scientific and philosophical approaches to theories of perception to date. A simple example: in a recent *Brain and Behavioral Sciences* article, Firestone and Scholl (2016) contended that “Cognition does not affect perception: Evaluating the evidence for ‘top-down’ effects.” Yet, the first sentence of the abstract reads: “What determines what we *see*?” (emphasis added). Keller’s (2016) commentary to the lead article in the same issue pointed out that the central claim of Firestone and Scholl may not apply to other sensory modalities, especially olfaction. Olfaction is a strongly evaluative sense in which perception and judgment are deeply entangled. There is a solid rationale not to circumvent the specificities of olfaction and other forms of chemoreception when it comes to a comprehensive theory of the senses.

Olfaction challenges key philosophical assumptions about how perception works, starting with the concept of “perceptual object.” In the philosophy of perception, the notion of olfactory objects is principally discussed as distal stimulus representation (Lycan, 2000, 2014; Batty, 2010a,b, 2013, 2014; Young, 2016; Millar, 2017). Perceptual objects, on this account, are either accurate or inaccurate mental representations of their physical causes. Nearly every philosopher here started with the understanding that olfactory objects refer to general qualitative categorizations that characterize a mental object, e.g., apple or skunk. Critics of this approach have pointed out that this conceptualization of olfactory objects, as representational of their sources, is heavily visuocentric and ignores the particulars of odor perception (Barwich, 2014, 2018; Smith, 2015; Keller, 2017). Disagreement between the two camps mainly concerned the nature of odors as mental representations; specifically, to what extent the chemical stimulus counts as the decisive factor for what is an accurate perceptual representation in olfaction. Smith (2017b), p. 793, summarized the core issue: “The grouping of odors in olfactory perception reflects the behavioral needs and responses of individuals, not physical similarities among the stimuli.”

Despite increasing philosophical interest in the sense of smell, the notion of olfactory objects received little examination concerning its biochemical basis and neural underpinnings. Would a naturalistic account of olfaction require revision of its philosophical treatment? Other philosophers have already advanced biologically informed perspectives on perception, yet again primarily with focus on vision (Matthen, 2005; Chirimuuta, 2015). As an example, Burge (2010) argued for a behavioral and evolutionary interpretation of perception concerning its purpose rather than accuracy. Olfactory scientists share this sentiment: “Recent research suggests that philosophical skepticism about the representational capacities of olfactory perception is a straw man, and more importantly, perhaps beside the point” (Castro and Seeley, 2014). Behavioral function, not idealized stimulus representation, characterizes olfactory perception: “Stimulus representation isn’t the primary business of olfaction. Rather, its job is solving a problem of valuation, rapidly encoding the biological salience of a stimulus and priming our multisensory representation of it to contextually appropriate action” (Castro and Seeley, 2014).

Nonetheless, these arguments do not challenge the underlying idea of perceptual objects as the starting point of the debate. Whether the notion of perceptual objecthood, traditionally framed by the workings of vision, applies to olfaction or requires revision needs examination. How fundamental is the idea of perceptual objects to theories of perception? Olfaction offers an excellent case to review the notion of perceptual objecthood and its role in theorizing about the senses against the backdrop of recent insights into the biological foundation of its perceptual processing.

This article examines the three criteria of perceptual objecthood in philosophical theories of smell: stimulus representation, perceptual constancy, and figure-ground segregation. The analysis shows that the traditional notion of perceptual objecthood does not apply to olfaction because the biological basis of odor coding is fundamentally different from the coding principles of the visual system. Perceptual objecthood is a concept used to analyze the identity of sensory input and especially its persistence and invariance. The identity of visual objects is contingent upon the sensory mechanisms that underpin feature extraction and integration. In olfaction, these mechanisms exhibit higher levels of variation, notably already at the periphery; so much so that the identity of olfactory sensations is shown to be inherently variable in its causal basis and does not comply with the idea of constancy in perceptual objecthood.

Specifically, Section “The Notion of Perceptual Objecthood and Its Explanatory Role” situates odors in recent philosophical debate on perceptual objecthood. Section “Three Criteria of Olfactory Objecthood” contrasts the coding principles in olfaction with vision to show that the three criteria of objecthood fail to be instantiated in odor perception. Olfaction, the argument concludes, affords perceptual categorization (the grouping of perceptual features under a label) without the need to form odor objects as representations of source objects.

## The Notion of Perceptual Objecthood and Its Explanatory Role

What constitutes a perceptual object and its explanatory centrality in theories of perception? Perceptual objects are routinely discussed under the premise of objectivity. Philosophical debate requires objectivity to demarcate the difference between appearance and reality in sensory perception. Scientific research is interested in objectivity to study the fundamental principles of sensory coding. Both are somewhat separate yet related endeavors that can be subsumed under one question, raised by Gibson (1966) and highlighted by Marr (1982), p. 29: “How does one obtain constant perceptions in everyday life on the basis of continually changing sensations?”

This view frames perceptual stability as the central function of the senses. Perceptual stability was never the strong suit of olfaction. The sense of smell has been traditionally dismissed as being a fickle, subjective sensation in the past. Champions of olfactory objects thus aim to show that they can warrant the sameness of olfactory perceptions under varying perspectives and conditions of stimulus exposure, demarcating perceptual objects from noise or subjective divergence (Lycan, 2000; Batty, 2010a,b; Young, 2016; Millar, 2017).

Three criteria of objecthood are evoked in support of this position. The first criterion features the external properties to which perception, as an objective representation of the real world, relates: stimulus topology. Vision facilitates the representation of three-dimensional objects constructed from predictable surface reflections of light. Analogously, odor objects are viewed as percepts corresponding to the molecular properties of the olfactory stimulus.

The second criterion is perceptual constancy, referring to viewpoint-invariant processing of sensory information. This criterion sanctions stable conditions to recognize a physical stimulus as the same object across varying circumstances. For example, despite variables such as shifts in surface reflections and our own movement, we identify visual objects across changing environmental conditions. This criterion is contingent upon the computational principles of the sensory system, extracting and integrating stimulus features into stable patterns. So the question to ask here is whether or how the organizational principles of the olfactory system facilitate perceptual constancy.

The third criterion of perceptual objecthood involves figure-ground segregation. Visual objects are discernible from general scenery or background. The same applies to smell; odors are discernible in comparison with each other. Odor objects should also be discernible with respect to their internal arrangement, i.e., as being composed of parts. How are these parts distinguished, or connect to the overall object? In other words, what determines the boundaries of parts and wholes in the perception of odor objects?

In sum, the determination of olfactory objecthood rests on three conditions:

1. stable representation of the physical stimulus as distinct perceptual classes,
2. perceptual constancy as perspective invariance, and
3. figure-ground segregation as whole-part discernibility.

These criteria were derived from general theories of perception originating in vision; in particular, representationalism (Lycan, 1996; Tye, 1997) and Gestalt psychology (Koffka, 2013/1935; Millar, 2017). Representationalism suggests a computationally stable link that correlates mental images with stimulus features; while Gestalt psychology assumes the presence of general principles by which configural mental images emerge from basic components.

The olfactory pathway and its characteristics are noticeably absent in the discussion of these criteria, despite being fundamental to their computation. Instead, the mechanisms of odor perception remain unspecified, even excluded from the philosophical analysis of olfactory objecthood. Cooke and Myin (2011) called this the “Independence Thesis”: the assumption that the mechanisms of a sensory system may produce perceptual objects, but that the details of their processing can be omitted in their study. So, even though olfaction shows physiological differences compared to other systems, perceptual analysis centers on percepts as the mental end products of their underlying biological processes. Whatever the biological details, what matters is access to their mental results. Therefore, the framing of objecthood builds on a stimulus-response model that blackboxes the system (Lycan, 1996; Young, 2016; Millar, 2017). That view is incorrect.

Odors are not unmediated perceptual expressions of chemical input because the isolated stimulus does not determine the content of its perception. Of course, the stimulus is a central element; yet, it is not the factor that determines *what* is perceived. That is the system that selects and filters features to be organized and integrated into sensory signals. This article shows that it is vital to understand how smell differs from vision (and, by extension, other senses). Other works have already pointed out that olfaction does not neatly fit the paradigm of vision, both in its perceptual and biological characteristics (Cooke and Myin, 2011; Barwich, 2014, 2016; Keller, 2017; Smith, 2017a,b). In one way or another, variations in the perception of smells constitute a measurable effect of the underlying causal processes (Wise et al., 2000; Barwich, 2014). The argument here details how biological differences in sensory processing mirror significant distinctions between olfaction and vision in their expression of perceptual content.

To set up comparative analysis of the olfactory system in the next section, a word on vision first. Traditionally, visual images have been modeled as the result of two separate subsystems: edge-detection and color. Here, edge-detection is responsible for the processing of spatial properties, where input signals from the retina are sent to the visual cortex *via* the thalamus to be hierarchically integrated into increasingly complex spatial detection-patterns such as lines, curves, and junctions (Hubel, 1988; Churchland, 1989). – Notably, the neural representation of edge-detection is topographic and stereotypic (i.e., the same in all members of a species). Later we hear that this distinguishes vision from olfaction. – It is edge-detection that usually is associated with visual object formation. Nonetheless, color vision is an integral part of this process, too. Older theories of perception considered color not to be constitutive of visual object formation, but to “add” to it in a kind of “coloring in” fashion (Livingstone and Hubel, 1988; comprehensive criticism of this model in Chirimuuta, 2015). Meanwhile, recent research called the strict division of edge-detection and color vision in visual object formation into question. Neurophysiological findings showed overlap in both pathways, early on in the periphery (Gegenfurtner and Kiper, 2003). Plus, color-coding is not only processing color but also resolving spatial features like edges (Mollon, 1989). Color is not merely contributing to but constitutive of visual object formation (Johnson et al., 2001; Kingdom 2003; Gheorghiu and Kingdom, 2007). Indeed, it is fair to say that the principles of the visual system are not as settled as commonly stated either.

What is the basis to examine olfaction in relation to vision then? Olfactory coding cannot be mapped one-to-one onto the characteristics of the visual pathway, and so analysis will engage with features of edge-detection and color vision to emphasize relevant differences with respect to overall perceptual integration. To compare perception in olfaction to vision thus must feature the coding processes that facilitate the identity and persistence of perceptual objects, regardless of whether these processes link to distinct, albeit overlapping, modules of a sensory system.

Overall, olfaction is highly susceptible to a number of causal factors: chemical, biological, and psychological. Setting up how these factors shape the perception of odors provides the background against which to evaluate the adequacy of the three philosophical criteria of objecthood in olfaction.

## Three Criteria of Olfactory Objecthood

Olfaction is a process that produces from volatile chemicals in the external world a perceptual impression that sometimes links to concepts (“cooked cabbage”) and, at other times, seems to lack a distinctly descriptive character (what *is* that smell?). It is quite common to describe smells with respect to their associated source objects. Still, in the words of Magritte, *This is not a pipe*. Odor labels are conceptual “proxies,” they should not be taken for the real thing (i.e., a perfumer can create a perceptual image, such as apple, from various molecules which need not be present in real apples). So we are advised to question:

What is the output of the process of olfaction; more specifically, what defines the content of olfactory experience? An answer is hard to come by because olfactory responses do not appear to be stable but vary a lot, between people but also in the same individual. Its variability is the reason why olfaction has long been excluded from theories of perception. It is also the reason why philosophical interest in olfactory theories was framed around the chemical stimulus as the stable element in the stimulus-response equation (Lycan, 2000, 2014; Batty, 2010a,b, 2013; Young, 2016; Millar, 2017). Here, the reasoning goes that if it can be argued that odors are (1) rule-based representations of the stimulus, and (2) shown to be constant, meaning perspective invariant, as well as (3) distinct in their elemental composition in comparison with other odors, then smells form perceptual objects.

Perceptual variability, on this account, is seen as distortions or illusions that occur when some factors interfere with the normal causal process of perceptual object formation. Hence, these variations are either special cases or deviations to be “explained away.” The norm is stable object perception, as in vision. The following subsections challenge this ontological premise of odor objecthood in the current philosophical debate. Specifically, it is shown that perceptual variation in olfaction is not a mark of subjectivity or distortion. It is the hallmark of olfaction as a sensory system to facilitate its central function: namely, detecting changes in the chemical composition the environment, not a context-invariant representation of an object (Köster et al., 2014; Barwich, 2018).

Variability is not the same as subjectivity (i.e., as having no objective measure) because variation in olfactory perception has an objective basis in the mechanisms of odor coding. Of course, this statement about objectivity as grounded in the sensory coding mechanisms applies to vision as well. Indeed, some variation also occurs in visual perception. That said the causal grounds and effects differ. Variation in vision routinely relates to higher-order processing. Consider the recent social media phenomenon of “the dress,” which was perceived as having different color combinations; people either saw it as being blue and black or white and gold. This effect is not related to visual receptor coding or sensitivities but linked to the computation of visual input in central processing. (Although, as a reviewer pointed out, there is suggestive evidence that this phenomenon may also relate to coding at the periphery; Rabin et al., 2016. Indeed, such shared grounds of variability should invite us to rethink the principles of vision.)

Still, at the periphery, the visual system is striking stable, discrete, and invariant in its conditions of feature extraction and integration. Meanwhile, the opposite is true in olfaction, as the following sections illustrate. In addition to higher-level integration and top-down effects (also known in vision), olfaction is highly variable and lacks discrete feature extraction as well as coordinated feature integration already at the periphery in receptor coding (different from vision). This difference results in numerous effects of perceptual variation that are not accommodated by the notion of objecthood as derived from theories of vision.

### Stimulus Representation

How do molecules result in mental images? Philosophical debate about olfactory objecthood centers on the chemical stimulus for its analysis of odor objects. Most have argued that odors can be accommodated under a physicalist account: just like color is said to correlate with physical features, the visible spectrum, odors correspond to molecular features (Lycan, 2000; Young, 2016; Millar, 2017). However, any stimulus-response model – regardless of modality – must be measured against the conditions of the sensory system.

There are two responses to a comparison of light and odor chemistry for stimulus-response models. First, such naive physicalism does not hold in color vision either (Matthen, 2005; Chirimuuta, 2015). Not all colors have a physical expression in wavelength. Consider pink, which is a computation of the brain (“white light minus green light”), not directly a feature of the world. Second, olfaction does not work like vision in its coding. Such coding differences are vital when it comes to the question of odor object formation. A comparison of visual with odor coding must address two issues in this context: what is the causal disposition of the chemical stimulus, and how does the stimulus interact with the sensory system (i.e., receptor coding).

Starting with the stimulus, the visual stimulus behaves in a predictable fashion: reflections of light rays in edge-detection have a precise angle. The same cannot be said about the chemical stimulus of smell: odorants are unpredictable in their environmental trajectory. What about the predictability of the coded molecular features in smell? Here, a comparison with color vision illuminates the difference. Color vision is low dimensional: it is determined by one key parameter, the length of electromagnetic waves. Meanwhile, odorants are multi-dimensional; they are characterized by about 5,000 molecular parameters (Keller and Vosshall, 2016).

Odor chemistry is strikingly complex. The olfactory stimulus is structurally highly irregular in how its molecular features link to specific perceptual effects. Minimal alterations in chemical composition, sometimes just of one carbon atom or methyl group, can result in substantive qualitative shifts (Rossiter 1996; Sell, 2006). Similarity in chemical structure does not necessitate similar odor quality: consider isosteric molecules, which are almost identical in shape and structure, yet have different odors. In contrast to the visual system, olfactory quality is not reducible to a linear stimulus-response model. This applies to individual odorants. It is rendered even more visible in mixture perception.

Commonly we do not perceive the smell of single odorants but mixtures. Molecules in a mixture regularly change their chemical properties and behavior, sometimes unpredictably. Unlike light in vision, the causal disposition of the olfactory stimulus can alter. Mixture perception does not operate by neat additive principles of stimulus combination. Consider coffee aroma, which consists of several hundred of chemicals. None of these elements, in isolation, smells of coffee. Many of these individual components have a robust individual odor, such as indole with its overpowering fecal scent. In coffee aroma, this fecal smell is not present but blends with the other components to form a qualitatively new aroma. Odor chemistry involves several other effects involved in the masking and blending of aromatic compounds.

There is much more to be said about stimulus chemistry, of course. Suffice to say, the computation of odor from molecular structure is not straightforward. Its solution remains unresolved, if not debatable in light of recent receptor studies (Poivet et al., 2016, 2018). The interaction with the receptors are what really determines the causal disposition of the stimulus in relation to the sensory system, meaning what kinds of information the sensory system is able to pick up and process. This brings us to the system selecting the input.

The biology of olfaction is not less complicated. It involves receptor coding as well as the organization of neural activity. This article will focus primarily on receptor biology for brevity (analysis of the neural organization of odor signaling in Barwich, 2018). The receptors alone give us plenty to talk about.

Differences to vision emerge immediately. The coding of primary colors, a central notion in visual perception, derives from the tuning of the three cones. To put this into perspective, human olfaction employs about 400 different receptor types. This contrast in number is not the only remarkable feature of the olfactory system. Olfactory receptors are also governed by different *coding principles* than vision.

Odor receptors operate by combinatorial coding and are highly variable in their tuning to molecular features. *Combinatorial* coding means that one receptor recognizes different features of different odorants; conversely, one odorant can be detected by several receptors (Malnic et al., 1999). In terms of *tuning*, some odor receptors detect a broader range of features and odorants than others (Poivet et al., 2016, 2018). What that means is that analogies to color vision break down: there is no set of primary smells coded by a set of receptors that, in their combination, explain the composition of other smells. Instead, there is a smorgasbord of possible olfactory stimuli and qualities (stimulus estimates range up to one trillion; Bushdid et al., 2014).

Back to the subject of odor object formation, these two factors (the mechanism of combinatorial coding, as well as variations and range in receptor tuning) carry two significant consequences for the perception of odor:

1. Combinatorial coding explains the vast amount of odors we can perceive, including the fact that we detect entirely new smells – immediately. It does not matter to the nose whether it encounters a molecule known to our ancestors, or an odorant created by a chemist in a laboratory 5 min ago. Your nose does not have to evolve a new set of receptors to do this. It picks up distinct features (e.g., the polar surface area of a molecule), which can occur in countless chemical permutations and is encoded by endless molecular combinations by the sensory system. For the identification of odor objects that means that there are odorants that did not exist in nature and have a quality that no human has smelled before, and which cannot be explained by reference to other odorants. In contrast with color coding, we cannot say: “Oh, the odor of this stimulus is computed as cis-3-hexenol minus hedione.”). Plus, these odorants may not have any known semantic object associated with them (like pee or rose). So, odor object coding must be understood in terms of receptor behavior. But receptor coding does not allow for straightforward stimulus-response mapping because:
2. Unlike vision, where the three cones are uniformly tuned to specific wavelengths, odor receptor coding is not uniform. Each of the 400 receptors operates by a different receptive range, its own receptor chemistry. The resulting causal mosaic of stimulus-receptor coding in olfaction makes it impossible to map stimulus activity (as represented by the sensory system) directly onto distinct features of physical stimulus space (Poivet et al., 2016, 2018). One could argue that vision also operates in a combinatorial way across the color cones (to facilitate the calculation of colors without an associated spectrum, like pink). However, there exists a crucial difference between vision and olfaction in their mechanism of combinatorial coding. In color vision, all photoreceptors respond to the same stimulus property, wavelength, while the olfactory receptors respond to about 5,000 different parameters in a non-linear, non-additive fashion (e.g., hydrophobicity, molecular weight, stereochemistry, volatility, etc.) (Ohloff et al., 2011). Consequently, feature extraction in olfaction is not homogenous; an issue highly relevant for signal integration and the question: which stimulus feature does a neural signal propagating receptor activation actually stand for?

What this difference amounts to is that, ultimately, odor qualities are determined by the constitution and coding of the receptors, not the chemical topology of the stimulus. It is worth highlighting here that experiments comparing stimulus chemistry with receptor behavior in the classification of odorants revealed significant differences in odorant grouping (Poivet et al., 2016, 2018). In other words: olfactory receptors arrange the chemical similarity of odorants by different features than an analytical chemist would model these molecules. The difference is fundamental; Poivet et al. (2018) showed that receptors responded to and grouped odorants by features that were not predicted by traditional stimulus chemistry (Keller et al., 2017). *Stimulus coding at the receptor level is not coextensive with the chemical topology of the distal stimulus.*

In further consequence, it is anything but apparent that olfaction affords a position of representationalism. Philosophical hand-waiving to “microstructure,” as the material basis for stimulus representation, does not lend plausibility to the question of olfactory objecthood. It is the receptors that count, not the stimulus. And the receptors do not afford a direct mapping of odor quality to molecular structure.

This view becomes indisputable once we take a closer look at receptor genetics. Receptor genetics is one of the central causes for variation in olfactory responses. With a few exceptions, people *typically* have the same set of visual cones. Olfaction is different. Each person has a unique expression of receptor patterns in their nose, *by default*. Does that imply that individual noses also smell the world differently? It looks that way! Recent studies linked the genetic diversity in odor receptors to variations in perceptual responses (Keller et al., 2007; Trimmer et al., 2019). On top of that, the vast number of olfactory receptor genes in the human genome allows for many mutations to take effect. Such mutations lead to notable differences in the experience of odor. For instance, cilantro (coriander) is intensely disliked by some people, who perceive its aldehydes as soapy and pungent instead of fruity and green. This perceptual effect stems from a genetic mutation near the olfactory receptor gene OR6A2 (Eriksson et al., 2012).

Next to differences in stimulus behavior, receptor coding and genetics, is receptor sensitivity. There are many odorants whose perception can vary substantially depending on receptor sensitivity. Take androstenone, a pig pheromone (Wysocki and Beauchamp, 1984). Androstenone smells differently to people with varying receptor sensitivities. Some people find it smells unpleasantly like urine, others perceive it as sweaty like body odor, and to others, it appears as woody, to a few it even comes off as vanilla or floral. Frankly, it is meaningless to ask whose perception is “accurate” or a correct representation. It is not a matter of subjectivity either, as this perceptual divergence grounds in distinct, measurable causal differences. These are only some examples of how biology, not isolated stimulus chemistry, determines odor perception.

What if we understood stimulus representation in terms of receptor patterns, instead of odor chemistry? This strategy was pursued by Batty (2010a,b) and, to some extent, Millar (2017) (conflating receptor coding with stimulus topology). Batty and Millar referenced Wilson and Stevenson’s (2006) biological definition of odor objects *via* receptor activation. Even in this modified account, the criterion of stimulus representation collapses. To explain this, it matters to disentangle the scientific notion of odor object from the philosophical concept. Wilson and Stevenson’s definition does not link odor objects as receptor patterns directly to perceptual categories (skunk, pee, and apple). The scientific use of odor object refers to the neural signals as the target of scientific explanation. Odor objects as receptor patterns determine the composition of the neural signal projected and organized throughout central processing by the olfactory system. This scientific notion is not co-extensive with the philosophical concept of odor object as defined above. (Additionally, Wilson and Stevenson emphasized the need to consider context-dependence in processes of learning, instead of stable stimulus-response coding, as the basis of olfactory perception.)

Identifying odor objects in the philosophical sense with combinatorial receptor patterns leads to conceptual pseudo-problems. Say, the epithelium of one person (P1) expresses four of five receptors coding for a specific odorant like hedione, while another person (P2) has all five. Do they perceive the same olfactory object? If not, the notion of odor object for the criterion of stimulus representation breaks down. If yes, we are ending up with a biological version of Theseus’ ship. Say, P1 expresses four of six receptors coding for an odorant {1 2, 3, 4}, while P2 also has four receptors but not the same set as P1, either {1, 2, 3, 5} or {1, 2, 5, 6}. Do they perceive the same olfactory object in this case? Moreover, does only one of these two options have the same odor object, or both, or neither? Such game of definitions could be played ad absurdum without ever arriving at real insights into the nature of odor perception or objecthood.

One may object that what counts is stability within rather than across individuals. However, individuals undergo alterations in their receptor repertoire, too. Receptor expression patterns do change during an individual’s lifetime with changes in exposure to odorants (Jones et al., 2008). This is not the case in vision.

The upshot is that variability in perceptual responses is not necessarily an expression of subjectivity, but mirrors the causal mechanisms of the olfactory system. Variations in sensory coding determine and explain deviations in perceptual content. What constitutes objectivity in perception, therefore, are the causal principles of the system, not philosophical armchair intuitions about stimulus representation. Variation is not to be explained away as it constitutes the fundamental *modus operandi* of the olfactory system.

### Perceptual Constancy

The ability to recognize and identify particular objects through time and space is perhaps the most convincing or intuitive criterion of perceptual objecthood. Its effects are salient in vision. The brain is an extraordinary organ in how it makes sense of visual input hitting the retina from all sorts of angles and constructing a tractable image that exhibits a constancy of features across changing perspectives and lighting circumstances. Next to variations of perspective are other challenges to visual processing; e.g., we can identify visual objects even if they are partially occluded, part of elaborate scenery, or distorted in some features.

This understanding of perceptual constancy applies to olfaction in Millar’s (2017) view: “we require more than a mere moment-by-moment individuation of odours; we also need these odours to retain their identity across change (involving object recognition capacities.” Millar’s central argument for perceptual constancy as a criterion for olfactory objecthood is that “[w]e experience odors as discrete units that bear properties and retain their identities through perspectival change.” Millar differentiates between two types of perceptual constancy: on the one hand, we see objects as “complete,” meaning we can identify perceptual objects even if some of their physical features are occluded. On the other hand, we can detect invariant features of a perceptual object despite shifts in perspective. These types of constancy imply that there are specific causal features that ensure stability in the mental representation of odor objects.

The problem with Millar’s argument is that it starts with an incorrect premise. Odors do not have a discrete identity retained throughout various perspectives, sensory and cognitive. Three examples should hammer this point home.

First, there is the mechanism of *selective adaptation* in olfaction (Hettinger and Frank, 2018). Prolonged exposure to an odor, especially of complex mixtures, alters one’s perception of its quality. The reason for that is, again, receptor behavior. Odor receptors habituate quickly to a stimulus. Moreover, olfactory receptors habituate and desensitize at different speeds. So if one smells an odor blend for a while, the receptors get desensitized to some of its stronger ingredients while others, to which receptors are still responsive, become more prominent in return. So, it is not clear what Millar alludes to as “discrete units.” (Selective adaptation is a serious impediment for olfactory psychophysics; a challenge visual scientists are lucky not having to deal with: You can expose a subject to a visual stimulus repeatedly, for several minutes, without fearing that this will affect what colors or shapes they perceive.) Even the sequence in which you administer odorants affects their perception: your perception of a cherry odor will exhibit a smoky note if you were exposed to a smoky odor beforehand, but not if you smelled the cherry odorant first (Cooke and Myin, 2011). One may object that in some cases at least (like the cherry one), the odor is still conceptually the same by presenting variations of a common theme (“cherry” plus something else). In reply, consider the next case.

Second, an olfactory mixture can, when perceived under altering perspectives, be experienced as having different conceptual categories. In a study by Herz and von Clef (2001), participants smelled identical pairs of five mixtures, each administered with different verbal labels (e.g., “parmesan” and “vomit” for butyric acid, or “musty basement” and “incence” for Patchouli). Subjects reported that the mixtures smelled different, although each pair of vials contained the same stimulus. The same stimulus can elicit a markedly different phenomenological experience, as well as qualitative category, depending on changes in the context of its exposure. Different semantic associations result in diverging perceptions of the same stimulus simply by experiencing an odor with a different conceptual tag. Indeed, descriptions of an odor vary significantly if a source object is visible or not; a fact that the German psychologist Hans Henning recognized as early as 1916 (Henning, 1916). Still, one may object to the example of Herz and von Clef (2001): since participants were not aware that these pairs were identical, they were simply “fooled” by the verbal labels. A third case will dissolve also this objection.

In a presentation at Columbia University in 2017, the master perfumer Christophe Laudamiel distributed smelling strips scented with sulfurol to an audience. People smelling it at first were uncertain regarding its quality. It smelled somewhat organic, perhaps a little meaty or sweaty, but also of something else. Laudamiel proceeded to show an image of warm milk. The audience murmured in agreement. Of course, it smelled like warm milk! Laudamiel continued by showing another image, this time of ham. The audience was startled. The strip with sulfurol suddenly smelled like ham. Laudamiel repeated his demonstration, alternating between the images of warm milk and ham. The perceptual switch continued to occur with the alternating images. The moral of the story is that, yes, we can recognize and assign a stimulus with general classes of odor qualities (like milk or ham). But the mere fact that our sensory system facilitates perceptual categorizations of input information does not mean that there is a discrete and constant perceptual object assigned to a stimulus.

In contrast with color vision, the border separating sensory input from cognitive judgment appears less categorical in smell than in vision. One reason for this is that the same olfactory stimulus, in different contexts, alters its perceptual meaning. Butyric acid can be perceived as Parmesan as well as vomit *because it is an element of both,* Parmesan and vomit aroma. Odor signals are ambiguous and underdetermined with respect to the conceptual identity of their source objects. This ambiguity resides on the periphery and does not constitute higher-level effects of illusions, distortions, or bias. Instead, variation in olfaction is explained by receptor coding and stimulus behavior in the environment. On the one hand, receptor coding: unlike visual cones, the olfactory receptors do not have a consistent, designated receptive range that picks out this, only this, and no other feature. Instead, they are broadly tuned and combinatorial. The signal the brain “sees” is only receptor activity in response to odorants, which may stem from several types of source objects. On the other hand, stimulus behavior: the stimulus itself is promiscuous in its occurrence; odorants frequently overlap in their occurrence in different mixtures and environments, so much so that they can get associated with various source objects of distinctly different conceptual identities. Therefore, odor identification is not poor (as argued by Young, 2018); it is conceptually underdetermined.

In conclusion, odors are not perspective-invariant like visual objects. Olfactory processing, in light of its inherent sensory underdetermination at the periphery, is fundamentally shaped by other sensory and higher-level cognitive processes (e.g., cross-modal cues, verbal descriptors, selective attention, or the mood of the perceiver).

Let us finish this section by applying these insights into odor biology to the criterion of perceptual constancy, as outlined in Millar (2017). Take the first type of perceptual constancy: completeness. It is not obvious what it even means for an olfactory object to be “complete.” Millar thinks of the unified experience of an odor. However, a complex, unified experience is not indicative of discrete perceptual objects (or migraines would meet that criterion; can a migraine be complete?). Plus, a unified sensory experience does not entail object recognition and identification. Otherwise, people would not struggle with identifying even familiar odors (the so-called “tip of the nose phenomenon” in Engen, 1991). In fact, integrated perceptual experience and object recognition (and identification) reflect distinct processing stages (also in vision, think agnosia).

The main point in need of attention is the second type of perceptual constancy: that perceptual objects have invariant features that allow them to be viewpoint independent. The reason why olfaction does not offer viewpoint independent perception is plain yet cannot be stressed enough: its coding differs from vision.

Consider how perceptual constancy is achieved in vision. Crucial to stimulus coding in the visual system is spatial representation (Hubel, 1988; Tootell et al., 1988). In parallel with the distinctly spatial coding of retinal input and its cortical projections, the perceptual representation of visual objects builds on specific spatial features, such as edges and boundaries, orientation, directionality, and extension (Churchland, 1989). Specifically, shape detection in vision builds on particular viewpoint invariant features in edge-detection, such as T-junctions defining the boundaries of objects and Y-junctions marking areas where surfaces join (Oram and Perrett, 1994). Based on these junctions, our visual system can calculate regularities in perspective, e.g., in the reconstruction of parallel structures. Optical illusions routinely build on this peculiarity in visual processing (e.g., the Ames room). In effect, the possibility of viewpoint invariant spatial features in vision is a result of the topographically distinct projection of signals from retinal cells sensitive to light contrast (center-surround cells) to cells in the primary visual cortex (and their hierarchical integration from simple cells to more complex cells) (Hubel, 1988).

What kinds of features are extracted by the olfactory system that might allow for feature-invariant representation? Feature extraction in edge-detection, as well as color vision, is much more confined and discrete compared to olfactory receptor tuning and combinatorics. In odor coding, there is no single definite parameter (like light contrast in edge-detection, or wavelength in color vision) that could operate as a viewpoint invariant feature. In a sense, the olfactory brain has to do some guesswork because odor receptors respond to several kinds of features as they are broadly tuned. Say, a receptor can be tuned to molecules with a particular polar surface area as well as odorants with atom groups in a specific orientation or a functional group (e.g., thiols). The interpretation of such underdetermined input signal thus depends on other, parallel signals. On a neural level, feature coding is not viewpoint invariant either.

Additionally, the neural representation of odors differs fundamentally from the topographic organization in vision. Over the past decade, it transpired that the olfactory cortex, specifically the piriform cortex, does not operate by a topographic principle similar to edge-detection (or even perceptual color maps) in the visual system (Stettler and Axel, 2009; Chen et al., 2014; Barwich, 2018). While research on rodents has shown temporal patterns associated with odors to emerge in the piriform (Roland et al., 2017), these patterns differ from the neural principles of the visual cortex: piriform patterns are sparse and require targeted, conditioned exposure to odorants; they are transient; and they are non-topographic and non-stereotypic. Thus, they represent contingent neural associations with an odor, not constancy in the way the visual system facilitates perspective-invariant perception through consistent and stable feature coding.

Overall, olfaction lacks a principal feature by which to calculate regularities or parallel structures like in vision, and as required for perceptual constancy if understood as perspectival invariance. In olfaction, we do not have a comparable set-up in its coding principles. While the olfactory system facilitates perceptual categorizations of stimulus information, it does not provide a comparable invariance in feature coding like edge-detection in vision. Notably, this applies also to the olfactory bulb, not only piriform cortex. The olfactory bulb is not a stereotypical but developmentally induced structure (Zou et al., 2009; also Belluscio et al., 2002; Feinstein et al., 2004). In even more exciting news, Noam Sobel has just reported a finding that “4.5% of all left-handed women in the #HCP data base have a perfectly normal sense of smell, but no olfactory bulb” (2019 meeting of the Association for Chemoreception Sciences, NeuroVero, 2019). While the implications of this finding are yet to be determined, clear is that this may revise understanding of odor coding and further differentiate it from vision.

Ultimately, the process of categorization (lumping information into groups) does not require the notion of objecthood.

### Figure-Ground Segregation

Figure-ground segregation allows the perceiver to pick out specific information against a noisy background. The cocktail party effect, referring to instances when one can hear someone saying their name in a chattering crowd, is an example of how the brain can filter and prioritize auditory information (Cherry, 1953). Olfaction, too, allows for figure-ground segregation. Smelling wine aroma, e.g., we can discern the presence of a light vanilla note against its general aromatic bouquet. Notwithstanding, Keller (2017) highlighted that there is an essential distinction in figure-ground segregation between vision and olfaction, one that further resonates with the argument on odor coding in the previous section: “Unlike visual perception, olfactory perception does not have a figure-background structure that reflects the relative position of stimuli in physical space.” The foregrounding of specific odor notes in wine aroma does not represent the arrangement of components in physical stimulus space.

Figure-ground segregation in olfaction has to do with stimulus saliency. Sommeliers evaluate a wine by focusing on distinct criteria of observational likeness with other wine profiles. (They can point to subtle flavor notes that the layperson may have missed but, after paying attention, is also able to perceive.) Olfactory experts can “dissect” a complex olfactory mixture in its qualitative notes as a result of extensive training and selective attention (Smith, 2007, 2017a; Croijmans and Majid, 2016; Barwich, 2017). During this process, wine tasters, as well as perfumers for that matter, have to search for olfactory qualities in a complex mixture. They are taking several steps of sniffing, comparing and evaluating their perception against cognitive templates because these fragrant notes in complex mixtures do not reveal themselves all at once (Smith, 2007; Todd, 2010). Figure-ground segregation in odor perception is not immediate; it is a process of perceptual grouping that involves sequential processing of attention and iterative interpretation. To be good at wine tasting requires perceptual expertise, acquired by a prolonged training of particular perceptual grouping techniques. Essentially, this expertise builds on the disposition to separate sensory information and focus on salient features. This principle of perceptual grouping likewise applies to naïve (untrained) smellers, who have to learn to assign perceptual classes and meanings to olfactory stimuli in order to form context-sensitive associations (Wilson and Stevenson, 2006; Köster et al., 2014; Barwich, 2018). – Test it yourself with wine tasting kits!

Essentially, figure-ground segregation is a process that facilitates perceptual categorization, not perceptual objecthood. A distinction introduced by Smith (2007) explains this difference. Smith, using the example of taste, distinguishes between, on the one hand, the causal disposition of a stimulus to elicit a certain (range of) perceptual qualities and, on the other hand, the perceptual expression of said causal features as a phenomenal quality. This distinction is crucial for understanding olfaction because the perceptual expression of an odor does not correlate undeviatingly with the causal disposition of its stimulus: First, the same stimulus can elicit different qualitative experiences in individuals (e.g., based on genetic differences). Second, a complex stimulus can be perceived differently in its qualitative notes if perceivers group its features differently. For example, two wine critics may describe the bouquet of a wine in separate qualitative terms simply because different features in the wine appear salient to them in their perceptual evaluation, and so they pay attention to different olfactory notes in their characterization (Smith, 2017a,b). That does not make the wine different. They might even come to the same conclusion (identifying the specific type of wine, its vineyard and year). But their perceptual sequence of evaluating the wine may differ. On this account, figure-ground segregation can result in varying yet equally accurate *perceptual groupings* of the same stimulus.

## Perceptual Categorization in Decision-Making Processes

It is instructive to think of perceptual object formation as just one example of how the senses act as perceptual systems. Some sensory systems may afford the formation of perceptual objects. But that does not say that the notion of perceptual objecthood is fundamental to the process of sensory perception in every modality. Rather, perception operates by the two principal processes of discrimination (same or different) and categorization (grouping of features). The coding of perceptual objecthood constitutes a special case in perceptual processing. Perceptual objecthood, if understood as stimulus representation linked to perceptual constancy, seems to require distinct coding principles at the periphery. The predominant focus on vision covered up the fact that object formation in this sense is not instantiated equally in other sensory systems, not even in all exteroceptive senses.

In this context, this article demonstrated that olfaction does not fit the three criteria of perceptual objecthood in philosophical debate. The olfactory pathway revealed to rely on substantially different coding principles in comparison to the visual system. This article analyzed these coding differences in their impact on perceptual content formation and expression. In particular, the high variability in the perceptual experience of odors was shown not to be a matter of distortion but how the olfactory system operates in its processes of feature extraction, combination, and integration. Perceptual analysis was shown to fail if it excludes the specifics of the sensory system.

To conclude, odors need not form a perceptual object to display general categories in their perception and for the olfactory system to fulfill its proper role. If one considers how robustly processes of learning, expectation, and cross-modal cues shape content formation in olfaction (Smith, 2012, 2017a; Barwich, 2018), it is clear that the olfactory system facilitates general categorizations of input without being built to represent perspective invariant objects in the same way as vision.

What primarily drives olfactory perception is not discrete, stable odor object formation but context-sensitive decision-making (Barwich, 2017, 2018). After all, the same chemical stimulus can occur in many different contexts, changing its causal disposition (e.g., in a mixture), as well as its meaning and value for the perceiver. It matters profoundly to the perceiver whether butyric acid is encountered as part of food (parmesan) or contaminants (vomit). The function of olfaction is to recognize changes in context and, in turn, affordance of the material stimulus (Barwich, 2018). Therefore, the same physical information of a stimulus can be grouped differently and matched against various cognitive templates. Its perceptual expression must be examined accordingly. Upon encountering olfactory information, the brain essentially has to decide on the grouping, salience, and value of olfactory input by matching this information with learned templates of odor categorization and other cues (cross-modal, verbal), all indicating the context in which a decision about the incoming olfactory information takes place.

Processes of perceptual categorization, as decision-making, do not to require the notion of olfactory objecthood. Smell is intentional in the sense that it provides information about materials in the world. Its content, however, is not a perceptual expression of olfactory objects but of grouping sensory input into qualitative classes. How perceptual categories are formed is the real question that scientific and philosophical theories of olfaction must tackle next.

## Author Contributions

The author confirms being the sole contributor of this work and has approved it for publication.

### Conflict of Interest Statement

The author declares that the research was conducted in the absence of any commercial or financial relationships that could be construed as a potential conflict of interest.
